# HIV-Tat Induces the Nrf2/ARE Pathway through NMDA Receptor-Elicited Spermine Oxidase Activation in Human Neuroblastoma Cells

**DOI:** 10.1371/journal.pone.0149802

**Published:** 2016-02-19

**Authors:** Roberta Mastrantonio, Manuela Cervelli, Stefano Pietropaoli, Paolo Mariottini, Marco Colasanti, Tiziana Persichini

**Affiliations:** Department of Science, University “ROMA TRE”, Rome, Italy; University of Messina, ITALY

## Abstract

Previously, we reported that HIV-Tat elicits spermine oxidase (SMO) activity upregulation through NMDA receptor (NMDAR) stimulation in human SH-SY5Y neuroblastoma cells, thus increasing ROS generation, which in turn leads to GSH depletion, oxidative stress, and reduced cell viability. In several cell types, ROS can trigger an antioxidant cell response through the transcriptional induction of oxidative stress-responsive genes regulated by the nuclear factor erythroid 2-related factor 2 (Nrf2). Here, we demonstrate that Tat induces both antioxidant gene expression and Nrf2 activation in SH-SY5Y cells, mediated by SMO activity. Furthermore, NMDAR is involved in Tat-induced Nrf2 activation. These findings suggest that the NMDAR/SMO/Nrf2 pathway is an important target for protection against HIV-associated neurocognitive disorders.

## Introduction

In the late phase of HIV infection, despite highly active antiretroviral (ARV) therapy, approximately 50% of patients develop neurological complications, collectively termed as HIV-associated neurocognitive disorders (HANDs) [1]. Although neurons are rarely infected by HIV-1, neuronal cell death is a common hallmark of HIV neuropathogenesis [2]. Thus, the cellular and viral toxic products that are generated from activated or infected cells may be indirectly responsible for neuronal loss [3]. Tat is a viral transcription factor that mediates the transactivation of HIV-1 replication and is one of several HIV-1 proteins that are most likely involved in triggering the changes associated with HIV infection. Tat can interact directly with neurons after being released into the extracellular space by macrophages or infected glia within the brain [4]. Previous studies have reported that, among other HIV-1 proteins, Tat leads to a dose-dependent increase in oxidative stress and to a decrease in intracellular glutathione in brain endothelial cells and various other cell types, including neuronal cells [5,6]. On the other hand, ARVs have been shown to be neurotoxic in both pigtail macaques and rats in vivo, an effect that is mediated by an accumulation of reactive oxygen species (ROS) [7].

A growing body of evidence links the polyamine spermine (Spm) catabolism to neurodegeneration, as observed in various *in vitro* and *in vivo* models [8–11]. Endogenous Spm is a ubiquitous cell component that is essential for normal cellular functions and growth [12,13]. Spm is directly oxidized by the flavoprotein spermine oxidase (SMO), producing spermidine, the aldehyde 3-aminopropanal (3-AP) and hydrogen peroxide (H_2_O_2_) [14]. These oxidative products may act as negative regulators of cell growth and survival. Indeed, in several neurodegenerative diseases, augmented polyamine catabolism results in the generation of H_2_O_2_ and of a number of reactive aldehydes that participate in the death of compromised tissues [11]. Our previous data have shown that Tat is able to induce ROS generation through an upregulation of SMO activity and to reduce cell viability in SH-SY5Y cell cultures, thus providing a link between Spm catabolism and HIV neuropathogenesis [5]. Interestingly, we have also observed that Tat-induced SMO activation (which leads to ROS generation and neurotoxicity) is mediated by the stimulation of NMDA receptor (NMDAR) [5].

ROS can trigger an antioxidant cell response through the transcriptional induction of oxidative stress-responsive genes [15]. To balance ROS levels and counteract their toxic effects, cells employ several antioxidant enzymes, including NAD(P)H:quinone oxidoreductase type 1 (NQO1), catalase (CAT), superoxide dismutase (SOD), heme-oxygenase (HO), glutathione peroxidase, thioredoxin, and peroxiredoxins. The balance between ROS generation and antioxidants is essential for normal cell function. The nuclear factor-erythroid 2-related factor 2 (Nrf2), a leucine zipper redox-sensitive transcription factor, is an important anti-oxidant gene regulator (for a recent special issue on Nrf2 see [16]). In normal, healthy conditions, Nrf2 is sequestered in the cytoplasm by a cytosolic regulatory protein, Keap1 [17]. However, during oxidative stress, Nrf2 translocates from the cytoplasm to the nucleus, heterodimerizes with small Maf proteins (sMaf) and sequentially binds to the promoter regions (antioxidant response elements (AREs), also known as electrophilic response elements (EpREs)) of many phase II detoxifying and antioxidant genes [15]. In neuronal cells, a clear interrelationship between Tat-mediated oxidative stress and Nrf2 activation is still lacking. Notably, neurons are the main cell type affected by ROS-mediated toxicity, and antioxidant levels in HIV-infected patients are altered, a situation that can lead to increased oxidative stress [18,19].

Here, we investigated the effect of Tat on Nrf2 activation in human neuroblastoma cells and studied the role of NMDAR and SMO on Tat-induced Nrf2 activation.

## Materials and Methods

### Materials

Chlorhexidine digluconate (CHL) solution, MK-801 hydrogen maleate (MK-801), N-methyl D-aspartic acid (NMDA), N-acetylcysteine (NAC), Dulbecco's modified Eagle's medium (DMEM), fetal bovine serum (FBS), 0.25% Trypsin–EDTA solution, and gentamicin solution 50 mg/ml were obtained from Sigma–Aldrich (Milan, Italy). Bradford reagent was obtained from Bio-Rad Italia (Milan, Italy). All chemicals were of analytical or reagent grade and were used without further purification. The ARP697 HIV-1 Tat-B protein (101 aa) was obtained from the Centre for AIDS Reagents, NIBSC HPA UK, supported by the EC FP6/7 Europrise Network of Excellence, the NGIN Consortia, and the Bill and Melinda Gates GHRC-CAVD Project, and was donated by FIT Biotech, Estonia, Dr. J. Karn.

### Cell Cultures and Treatments

SH-SY5Y human neuroblastoma cells were purchased from ATCC (Manassas, VA, USA). As described in detail previously [5], cells were cultured in DMEM supplemented with 10% FBS and 40 μg/ml gentamicin at 37°C in a humidified 5% CO_2_ incubator. Confluent monolayers of SH-SY5Y cells were subcultured by conventional trypsinization. For the experiments, 3x10^5^ or 2x10^6^ cells were seeded in 35 -or 100-mm tissue culture dishes, respectively, and grown up to 80% confluence for 18–24 h before treatments. Working solutions of Tat were freshly prepared in culture medium from stock solutions stored at -80°C. Where indicated, SH-SY5Y cells were treated with 200 ng/ml HIV-1 Tat recombinant protein or NMDA agonist (1 mM) in serum-free DMEM. For the pretreatment experiments, either CHL (0.01 μM for 16 h) or MK-801 (10 μM for 2 h) added in DMEM supplemented with 10% FBS and 40 μg/ml gentamicin.

### Determination of SMO Enzyme Activity

As described in detail previously [5], the SMO polyamine oxidase activity was assayed using a modification of the chemiluminescence analysis reported by Wang et al. [20]. Luminol-dependent chemiluminescence was determined using a Lumat LB 9507 G&G Berthold luminometer. Luminol was prepared as a 100 mM stock solution in DMSO and diluted to 100 μM with H_2_O immediately before use. Cell extracts (1×10^6^ cells/sample) were assayed in 83 mM glycine buffer (pH 8.3), 20 μg/ml horseradish peroxidase, 0.2 mM 2-bromoethylamine (catalase inhibitor), 15 μM deprenyl (copper-containing amine oxidase inhibitor), 0.15 μM clorgyline (mitochondrial oxidase inhibitor), and 500 μM Spm as substrate to determine SMO activity. All reagents, with the exception of substrate, were combined and incubated for 5 min at 37°C, then 5 nmol luminol was added and incubated again at 37°C for 2 min. The sample was then transferred to the luminometer, Spm was added, and the resulting chemiluminescence was integrated over 40 s. Polyamine concentration was determined as described in Mates et al. [21].

### Quantitative Real-Time Reverse Transcription–Polymerase Chain Reaction (RT-qPCR)

Total RNA was purified by using TRIzol^®^ Reagent (Life technologies Italia-Invitrogen, Monza, Italy) and reverse transcribed into cDNA with GoTaq 2-step RT-qPCR system (Promega Italia Srl, Milan, Italy). cDNA was amplified for the NQO1 gene (fwd 5’-ATG TAT GAC AAA GGA CCC TTC C -3’ rev 5’-TCC CTT GCA GAG AGT ACA TGG -3’), CAT gene (fwd 5’-TCA GGT TTC TTT CTT GTT CAG-3’ rev 5’-CTG GTC AGT CTT ATA ATG GAA TT -3’), SOD1 gene (fwd 5’-AGT AAT GGA CGA GTG AAG G-3’ rev 5’- GGA TAG AGG ATT AAA GTG AGG A-3’), SOD2 gene (fwd 5’- AAT GGT GGT GGT CAT ATC A-3’ rev CCC GTT CCT TAT TGA AAC C-3’), and HO-1 gene (fwd 5’-CGG GCC AGC AAC AAA GTG-3’ rev 5’-AGT GTA AGG ACC CAT CGG AGA A-3’).

GAPDH mRNA (fwd 5’-TTG TTG CCA TCA ATG ACC C -3’ rev 5’- CTT CCC GTT CTC AGC CTT G-3’) was examined as the reference cellular transcript. PCR product quantification was calculated by applying the SYBR-Green method. Reactions were performed in a Rotor gene 6000 machine (Corbett research) using the following program: 45 cycles of 95°C for 15 sec, 60°C for 60 sec, 72°C for 20 sec. GAPDH mRNA amplification products were present at equivalent levels in all cell lysates. The data are calculated relative to the internal housekeeping gene according to the second derivative test (delta–delta Ct (2^-ΔΔCT^) method).

### Preparation of Nuclear Extracts

After treatments at the indicated time points, the cells were mechanically detached with a scraper in cold PBS. Nuclear extracts were prepared by adding buffer A (10 mM Hepes, 10 mM KCl, 1.5 mM MgCl_2_, 0.5 mM DTT, 0.1% NP40, protease inhibitor cocktail) to the cell pellets to separate nuclei from cytosol. After incubation for 10 min on ice and subsequent centrifugation at 12000 rpm for 10 min at 4°C, pellets containing nuclear fractions were resuspended in buffer C (20 mM Hepes pH 7.9, 420 mM NaCl, 1.5 mM MgCl_2_, 25% glycerol, 1 mM EDTA, 1 mM EGTA, 0.5 mM DTT, 0.05% NP40, 1 mM PMSF, 10 μg/ml aprotinin, and 10 μg/ml leupeptin) and incubated on ice for 30 min. A final centrifugation at 14,000 rpm was carried out, and the supernatants were collected, quickly frozen in liquid nitrogen and stored at -80°C. The total protein content of nuclear extracts was determined according to Bradford method [22].

### Analysis of Nrf2 Activation by Western Blotting

Equal amounts (40 μg proteins/sample) of nuclear extracts were subjected to electrophoresis in an 8% polyacrylamide gel and transferred to nitrocellulose membranes. Membranes were blocked with 5% non-fat dry milk for 1 hour and incubated overnight at 4°C with a polyclonal anti-Nrf2 antibody (1:1000; Abcam Italy, Prodotti Gianni S.p.A., Milan, ITALY) or with a polyclonalanti-laminB1 (1:4000, Abcam Italy). Lamin B1 was used as the reference protein amounts for nuclear lysates. Secondary peroxidase-labeled anti-rabbit IgG antibody (1:10000) was from Bio-Rad Italia (Milan, Italy). Detection was performed using ECL Western blotting detection reagents (GE Healthcare, Milan, Italy).

### ELISA-Based Measurement of Nrf2 Activity

The TransAM Nrf2 Kit (Active Motif, Vinci-Biochem, Firenze, Italy) was used to assay the DNA-binding activity of Nrf2 in the nuclear extracts. Ten micrograms of nuclear extracts from each sample in duplicate were incubated in a 96-well plate that was coated with oligonucleotide containing a consensus binding site (5’-GTCACAGTGACTCAGCAGAATCTG-3’) for Nrf2. For competitive binding experiments, which measure the specificity of the assay, 10 μg of nuclear extracts was assayed in the presence of wild-type or mutated competitor oligonucleotides. After 1 h of incubation, the wells were washed and incubated with Nrf2 antibody (1:1000) for 1 h at room temperature without agitation. Afterwards, the wells were incubated with HRP-conjugated antibody (1:1000) for 1 h at room temperature. Afterwards, a developing solution was added for 10 min. Finally, a stop solution was added into the wells, and the absorbance was read at 450 nm with a reference wavelength of 655 nm using a plate reader.

### Statistical Analysis

All data are expressed as the mean ± standard error of the mean (SEM) of n observations. Statistical analysis was performed by one-way ANOVA and subsequently by Bonferroni post-tests. Differences are considered statistically significant at p≤0.05.

## Results and Discussion

Free radical production and oxidative stress play important roles in the pathogenesis of various neurodegenerative diseases, including HANDs [23]. Numerous studies have shown the ability of HIV-1 viral proteins (gp120 and Tat) to increase nitrosative and oxidative stress in the brain [24,25]. Recently, we reported that HIV-1 Tat can induce oxidative stress and neuronal cell death through the production of H_2_O_2_ by a mechanism involving both polyamine metabolism and NMDA receptor activation [5].

**Fig 1:**
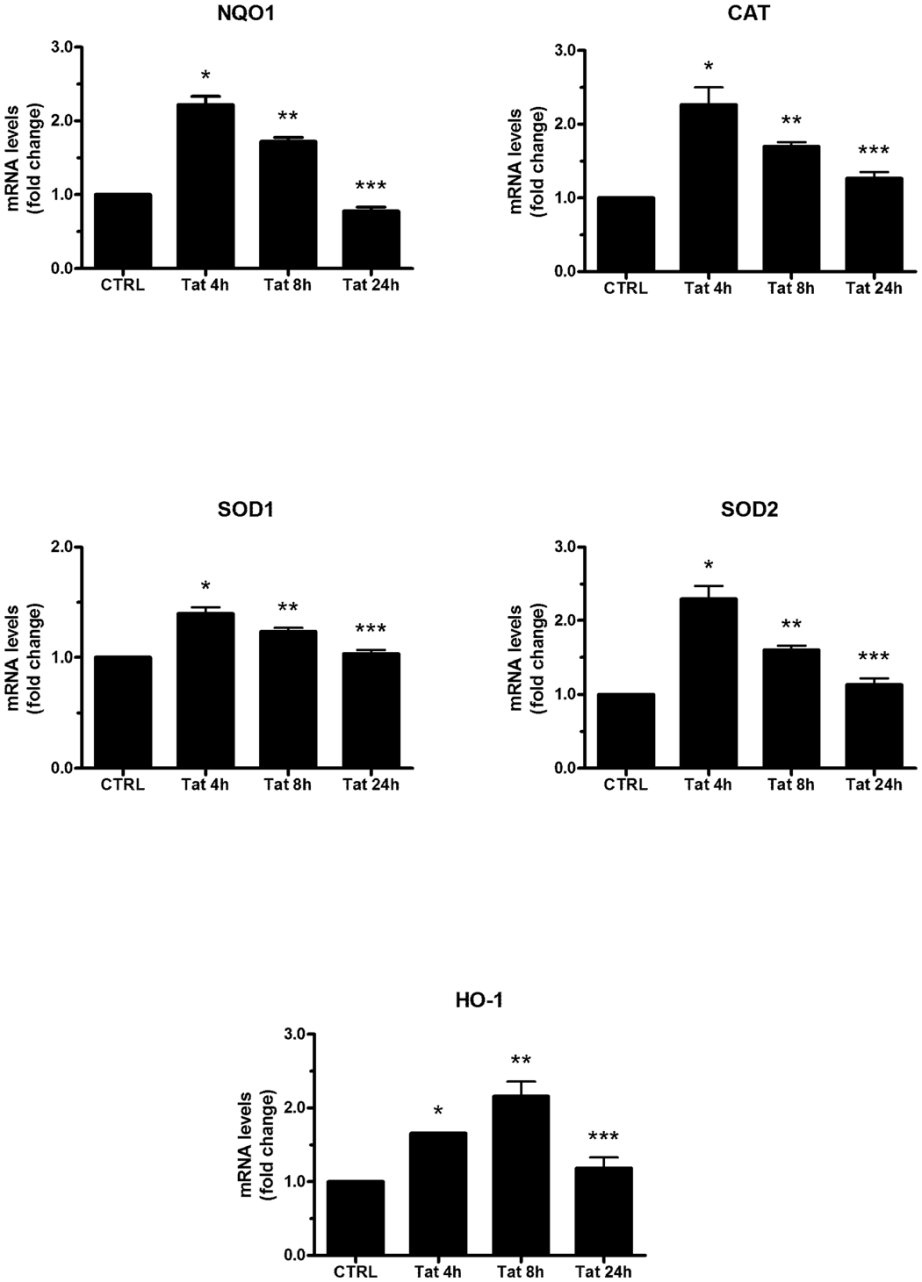
Effects of Tat on ARE-driven gene expression in SH-SY5Y cells. Cells grown on 35-mm-diameter tissue culture dishes were treated with Tat (200 ng/ml) for 4, 8, and 24 h. After incubation at 37°C, the cells were homogenized and total RNA has been purified to assess mRNA levels of several genes (NQO1, CAT, SOD1, SOD2, HO-1) by RT-qPCR. Data are calculated relative to the internal housekeeping gene (GAPDH) and are expressed as the mean fold change compared with control. Each value represents the mean ± SEM of three independent experiments. One-way ANOVA, followed by Bonferroni's test, was used to determine significant differences. NQO1: * p≤0.01 vs CTRL, ** p≤0.01 vs CTRL, *** Not significant vs CTRL; CAT: * p≤0.01 vs CTRL, ** p≤0.05 vs CTRL, *** Not significant vs CTRL; SOD1: * p≤0.01 vs CTRL, ** p≤0.05 vs CTRL, *** Not significant vs CTRL; SOD2: * p≤0.01 vs CTRL, ** p≤0.05 vs CTRL, *** Not significant vs CTRL; HO-1: * p≤0.05 vs CTRL, ** p≤0.01 vs CTRL, *** Not significant vs CTRL.

In oxidative stress conditions, however, many cell types activate an endogenous anti-oxidant response, which is mediated by the induction of phase II detoxifying genes and antioxidant genes. Here, we found that the treatment of human SH-SY5Y neuroblastoma cells with 200 ng/ml recombinant Tat (101 aa) for 4, 8, and 24 h was able to increase the mRNA expression of enzymes, such as NQO1, CAT, SOD1, SOD2 and HO-1. As shown in Fig 1, we observed an increase of mRNA expression of all the genes analyzed, reaching the maximum at 4 h (8 h for HO-1) after Tat treatment. At 24 h post-treatment, gene expression was reverted to nearly control levels (Fig 1).

Recently, another HIV-1 protein, reverse transcriptase (RT), was shown to induce an antioxidant response [26]. In particular, RT increased the transcription of the phase II detoxifying enzymes NQO1 and HO-1 in human embryonic kidney cells, revealing a direct link between the propensity of the viral proteins to induce oxidative stress and their immunogenicity [26]. Furthermore, it has been reported that gp120 significantly upregulates HO-1 and NQO1 in human astrocytes, suggesting a possible role of the antioxidant defense mechanism in promoting cell survival [27]. Notably, a progressive increase in serum catalase activity has been detected in advancing HIV infection, reflecting and/or compensating for systemic glutathione and other antioxidant deficiencies in HIV-infected individuals [28].

**Fig 2:**
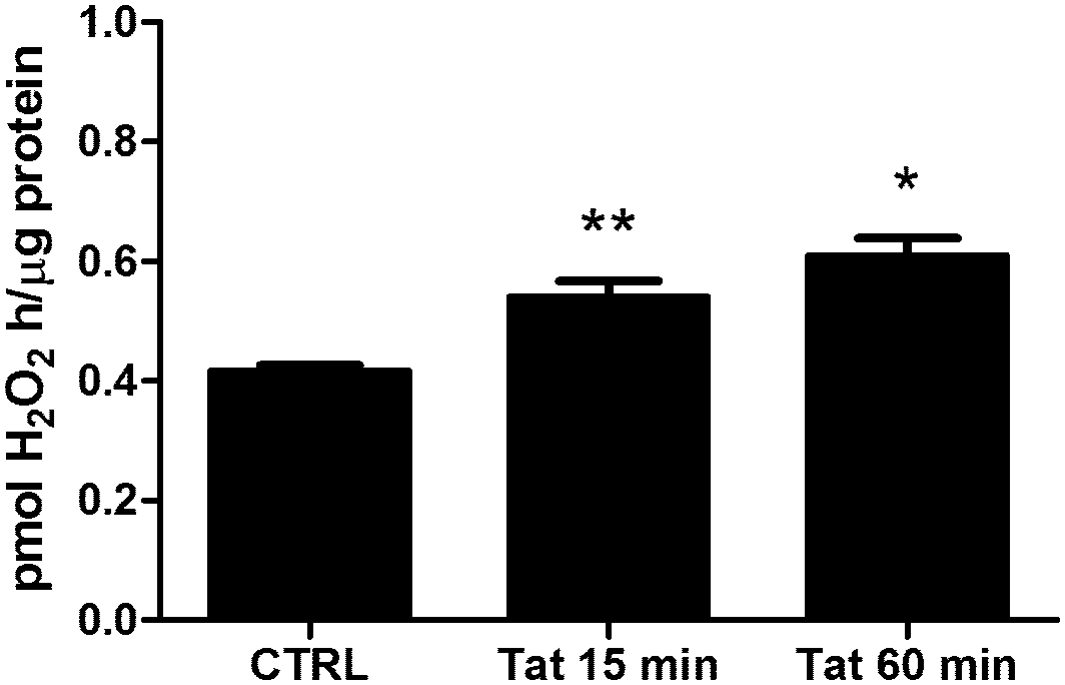
Effects of Tat on spermine oxidase activity. SH-SY5Y cells (1×10^6^) were treated with Tat (200 ng/ml) for 15 and 60 min. At the end of incubation cell extracts were prepared as described in Materials and methods and analyzed for SMO activity. The data shown are the means of three independent experiments. One-way ANOVA, followed by Bonferroni's test, was used to determine significant differences. ** p≤0.05 vs CTRL, * p≤0.01 vs CTRL.

**Fig 3:**
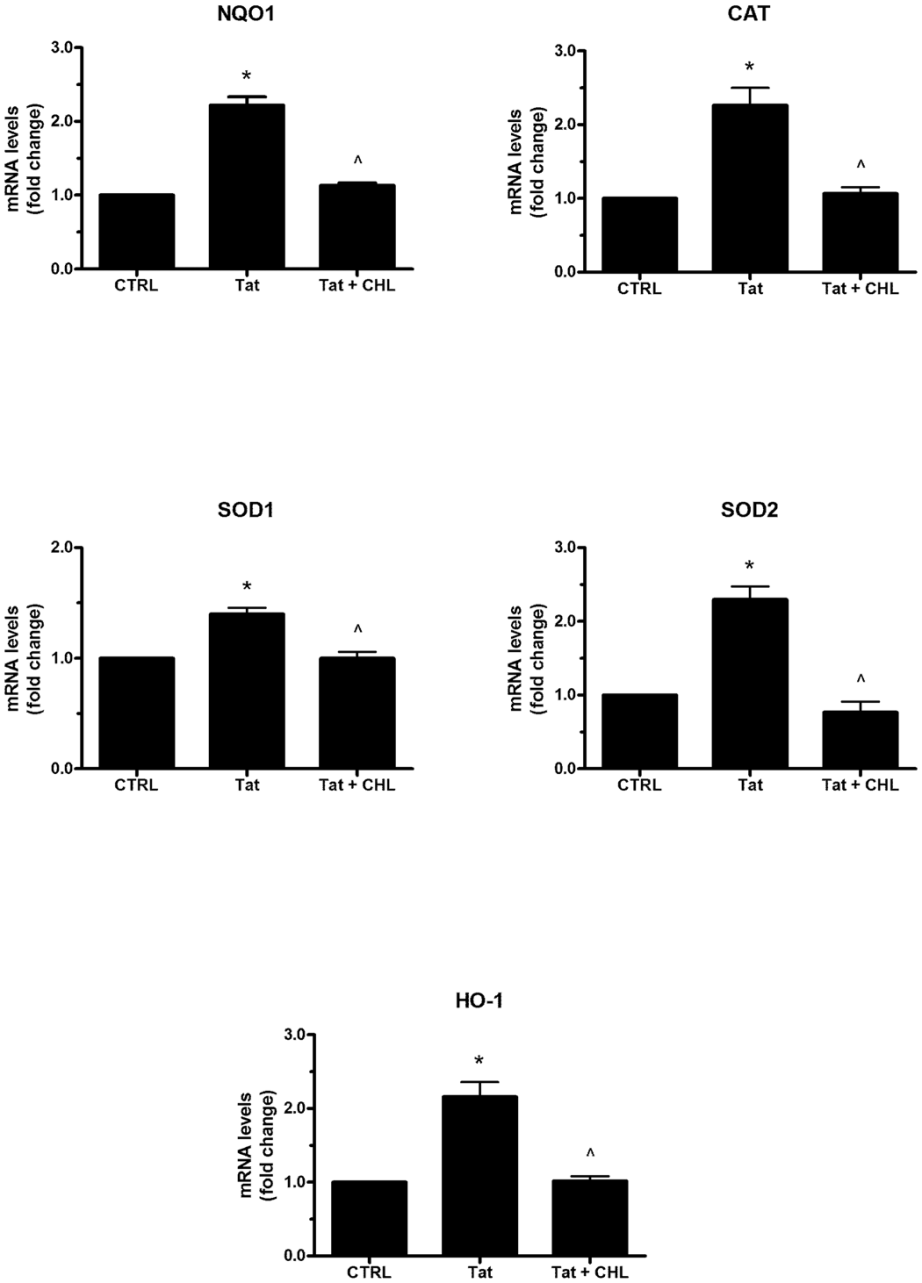
Effects of SMO inhibition on Tat-induced gene expression in SH-SY5Y cells. Cells grown on 35-mm-diameter tissue culture dishes were overnight pretreated with CHL (10 nM) or medium alone before the addition of Tat (200 ng/ml) for 4 h. After incubation at 37°C, the cells were homogenized and total RNA has been purified to assess mRNA levels of NQO1, CAT, SOD1, SOD2, HO-1 genes by RT-qPCR. Data are calculated relative to GAPDH and are expressed as the mean fold change compared with control. Each value represents the mean ± SEM of three independent experiments. One-way ANOVA, followed by Bonferroni's test, was used to determine significant differences. NQO1: * p≤0.01 vs CTRL, ^ p≤0.01 vs Tat; CAT: * p≤0.01 vs CTRL, ^ p≤0.01 vs Tat; SOD1: * p≤0.01 vs CTRL, ^ p≤0.01 vs Tat; SOD2: * p≤0.01 vs CTRL, ^ p≤0.01 vs Tat; HO-1: * p≤0.01 vs CTRL, ^ p≤0.01 vs Tat.

Normally, the expression of antioxidant enzymes is induced in response to oxidative stimuli, including ROS production. As we reported elsewhere, the treatment of SH-SY5Y cells with Tat for 1 and 4 h was able to induce ROS generation by the upregulation of the SMO enzyme activity [5]. Here, we hypothesize that Tat can elicit an antioxidant response in neuronal cells through a SMO-dependent activity. To address this question, we first assessed the ability of Tat to upregulate the activity of SMO at an early time point (i.e., 15 min). Therefore, a chemiluminescence analysis was performed to measure H_2_O_2_ production in extracts from cells treated with Tat (200 ng/ml) for 15 and 60 min. As shown in Fig 2, the SMO activity was already increased at 15 min post-Tat treatment; this effect was maintained for up to 60 min. Next, we wanted to determine whether the expression of detoxifying and antioxidant genes in response to Tat treatment was elicited by the SMO-induced activity. Therefore, we treated SH-SY5Y cells with 10 nM chlorhexidine digluconate (CHL), a strong competitive inhibitor of SMO [29], for 16 h before treatment with Tat (200 ng/ml). As expected, CHL completely prevented Tat-induced up-regulation of all the genes analyzed (Fig 3), thus suggesting that Tat may elicit an antioxidant response in neuronal cells through the activation of SMO.

**Fig 4:**
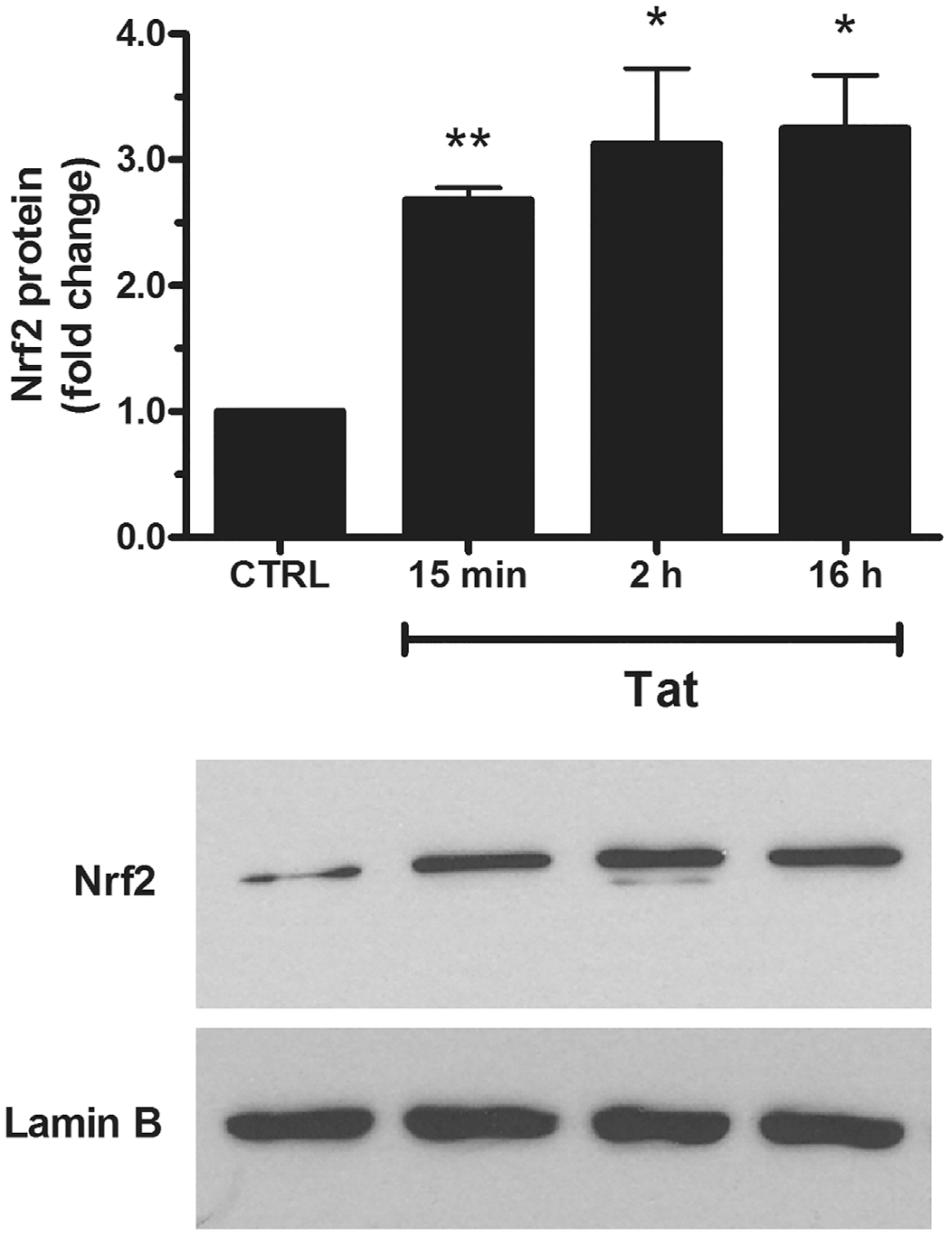
Effects of Tat on Nrf2 nuclear translocation in SH-SY5Y cells. Cells grown on 100-mm-diameter tissue culture dishes were treated with Tat (200 ng/ml) for the indicated time points. After incubation at 37°C, cells were mechanically harvested, and the nuclear extracts were prepared as specified in the Materials and Methods section to assess Nrf2 levels by western blot analysis. The histogram shows the densitometric analysis of the western blots for each sample. Values are calculated relative to the nuclear Lamin B content and are the means ± SEM from three separate experiments, each performed in duplicate. One-way ANOVA, followed by Bonferroni's test, was used to determine significant differences. ** p≤0.05 vs CTRL, * p≤0.01 vs CTRL.

Considering that ARE genes are mainly regulated by Nrf2, we investigated whether Tat was able to activate this transcription factor in human neuroblastoma cells. In particular, SH-SY5Y cells were treated with Tat (200 ng/ml) for 15 min, 2 h, and 16 h, and the levels of Nrf2 were measured in nuclear extracts by using Western blot analysis. As shown in Fig 4, Tat induced a 2.68-fold increase of the nuclear Nrf2 levels already at 15 min post-treatment, which was maintained up to 16 h. The effect of Tat on Nrf2 activation was also confirmed by a TransAm kit based on ELISA method. We found, for the first time, that Tat activates Nrf2 in neuronal cells, and our results are consistent with data reporting that Tat enhances the cellular expression of Nrf2 at the transcriptional and protein levels in MAGI cells [30]. Moreover, it has been reported that Nrf2 is also upregulated in response to gp120 in primary astrocytes, thereby suggesting a possible protective role of gp120-induced Nrf2 in regulating the levels of pro-oxidative and pro-inflammatory molecules in HANDs [27].

**Fig 5:**
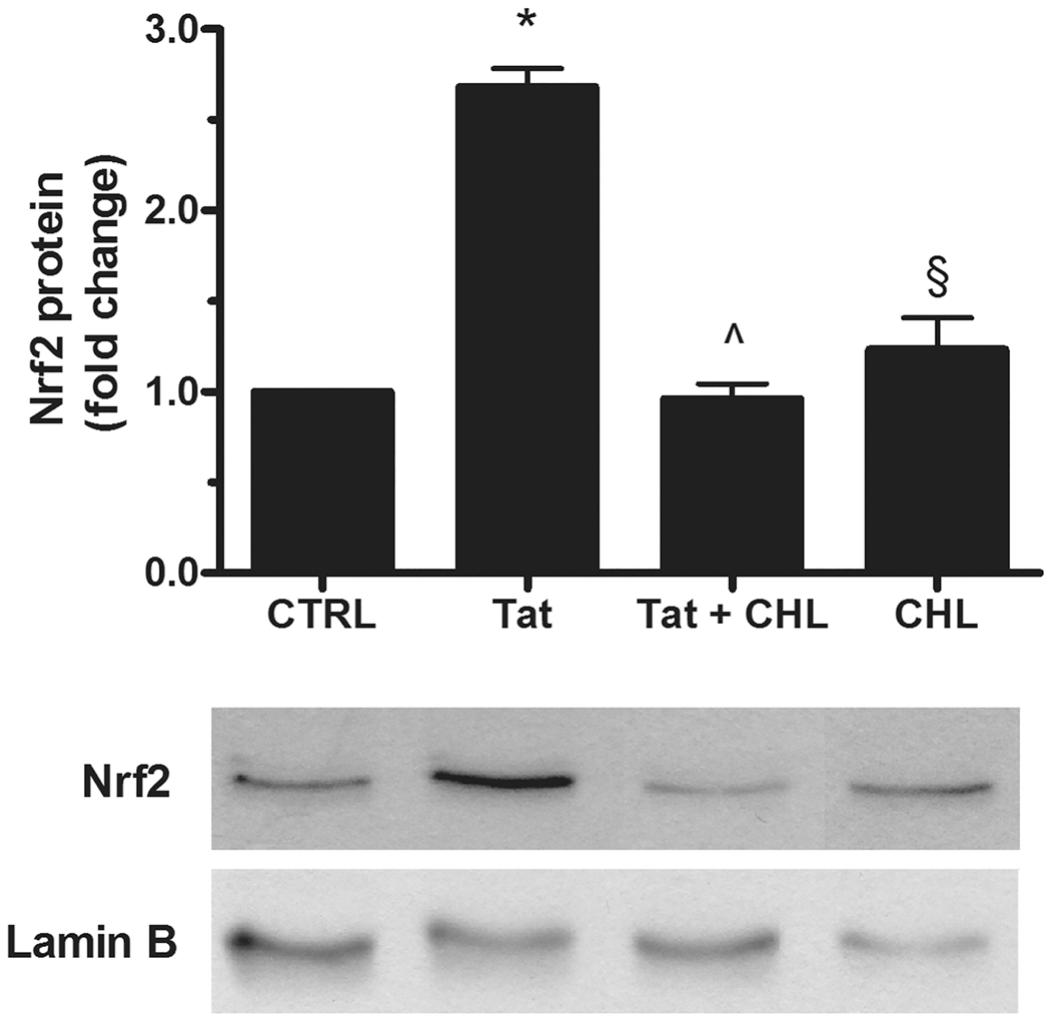
Effects of SMO inhibition on Tat-induced Nrf2 activation in SH-SY5Y cells. Cells grown on 100-mm-diameter tissue culture dishes were overnight pretreated with CHL (10 nM) or medium alone before the addition of Tat (200 ng/ml) for 15 min. After incubation at 37°C, cells were mechanically harvested, and the nuclear extracts were prepared as specified in Methods to assess Nrf2 levels by western blot analysis. The histogram shows the densitometric analysis of the western blots for each sample. Values are calculated relative to the nuclear Lamin B content and are the means ± SEM from three separate experiments, each performed in duplicate. One-way ANOVA, followed by Bonferroni's test, was used to determine significant differences. * p≤0.01 vs CTRL, ^ p≤0.01 vs Tat, § Not significant vs Tat + CHL.

As described above (see Fig 2), Tat is able to upregulate the activity of SMO at an early time point (i.e., 15 min). To investigate on the role of SMO in Tat-elicited Nrf2 activation, SH-SY5Y cells were exposed overnight to 10 nM CHL before treatment with Tat (200 ng/ml) for 15 min. Then, a western blot analysis was performed on nuclear extracts using an anti-Nrf2 specific antibody. As shown in Fig 5, the pretreatment of SH-SY5Y cells with CHL completely prevented Tat-induced Nrf2 nuclear translocation, thereby suggesting an involvement of SMO in this mechanism.

**Fig 6:**
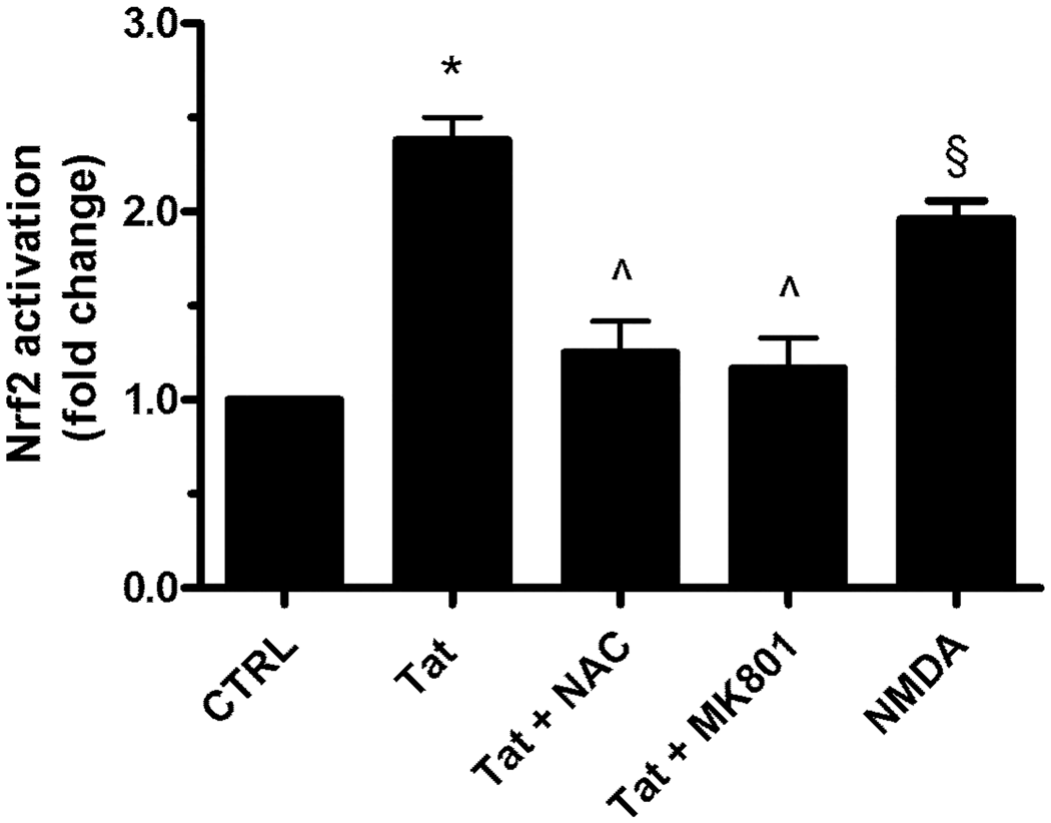
Role of NMDA receptor in Tat-induced Nrf2 activation. SH-SY5Y cells grown on 35-mm-diameter tissue culture dishes were pretreated for 2 h with MK-801 (10 μM), NAC (2 mM), or medium alone before the addition of Tat (200 ng/ml) for 15 min. After incubation at 37°C, the cells were homogenized, and Nrf2 activation was quantified by TransAM assay as detailed in the Materials and Methods section. Data points are the means ± S.E.M. from 3 separate experiments, each performed in duplicate. One-way ANOVA, followed by Bonferroni's test, was used to determine significant differences. * p≤0.01 vs CTRL, ^ p≤0.01 vs Tat, § p≤0.01 vs CTRL.

Previously, we reported that Tat was able to induce ROS production through the stimulation of SMO activity in neuroblastoma cells [5]. Here, we evaluated the role of SMO-dependent ROS generation in Tat-induced Nrf2 activation by pretreating SH-SY5Y cells with the antioxidant NAC, which is able to prevent Tat-induced ROS generation [5]. As shown in Fig 6, the pre-treatment of cells with NAC (2 mM) for 1 h before treatment with Tat (200 ng/ml) for 15 min inhibited Nrf2 activation.

As we observed elsewhere, Tat induces SMO activity and ROS production through the stimulation of NMDA receptor in neuroblastoma cells [5]. To investigate whether NMDAR was involved in Tat-induced Nrf2 activation, we pretreated SH-SY5Y cells for 2 h with the NMDAR antagonist MK-801 (10 μM) and then treated with Tat (200 ng/ml) for 15 min. Fig 6 shows the strong inhibitory effect of MK-801 on the activation of Tat-induced Nrf2. Consistently, NMDAR induced Nrf2 activation (Fig 6), clearly indicating that the stimulation of NMDAR can be responsible for the observed Nrf2 activation in neuroblastoma SH-SY5Y cells. Altogether, these results indicate that Tat induces the Nrf2 pathway through NMDAR-elicited SMO activation in human neuroblastoma cells.

**Fig 7:**
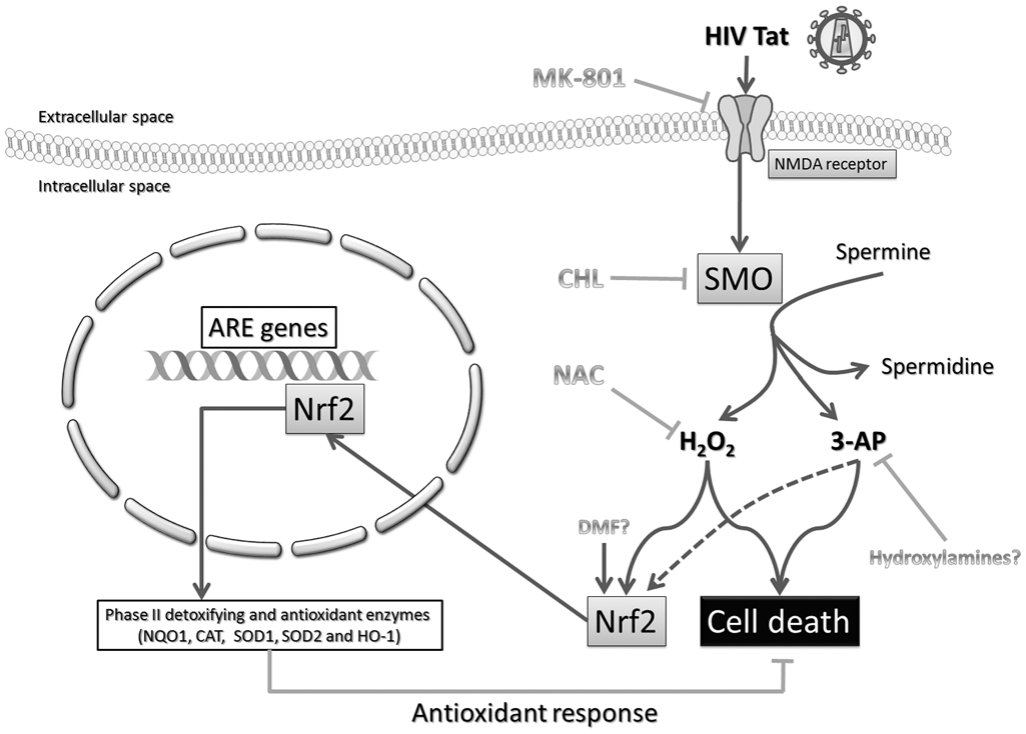
Proposed model of the role of NMDAR-elicited SMO activation in the Tat-induced Nrf2 pathway. On the one hand, HIV-1 Tat induces neuronal cell death through the production of H_2_O_2_ and 3-AP by a mechanism involving NMDAR-induced SMO activation. On the other hand, the same pathways are able to trigger an antioxidant response through the transcriptional induction of Nrf2-dependent ARE genes. For more details see text. Abbreviations: 3-AP, aldehyde 3-aminopropanal; ARE, antioxidant-response element; CAT, catalase; CHL, Chlorhexidine digluconate; DMF, dimethyl fumarate; H_2_O_2_, hydrogen peroxide; HIV, human immunodeficiency virus; NQO1, NAD(P)H:quinone oxidoreductase type 1; HO, heme-oxygenase; NAC, N-acetylcysteine; NMDA, N-methyl-D-aspartate; Nrf2, nuclear factor erythroid 2-related factor 2; SMO, spermine oxidase; SOD, superoxide dismutase.

The question now is whether the modulation of NMDAR/SMO/ROS/Nrf2/ARE pathways is sufficient for protection against Tat-induced oxidative stress (see Fig 7 for a schematic overview). As we described elsewhere, NMDAR/SMO/ROS activation leads to a weak (approximately 30%) neurotoxicity in Tat-treated SH-SY5Y cells [5], so, cell death occurs (although weakly) despite the activation of Nrf2/ARE pathway. A similar paradox has been reported by Akay et al. [7] in a study on the effects of ARV drugs in the central nervous system. In particular, the neuronal damage and death that occur following exposure to ARV drugs, despite the endogenous antioxidant response, suggest that this response may be insufficient or too delayed to protect cells from Tat toxicity [7]. Accordingly, Zhang et al. [30] report that Nrf2 activation induced by Tat in MAGI cells is not sufficient for protection. It is important to take into account that the activation of an antioxidant response is not only regulated by the induction of Nrf2 but also by post-induction responses that tightly control Nrf2 activation and repression back to the basal state, finally ‘switching off’ Nrf2-activated gene expression [15]. Notably, HIV-1 induces accelerated aging, and the redox imbalance may actively promote senescence [31]. Compared to age-matched controls, HIV-1 transgenic rats have been shown to have a significant reduction in the protein levels of Nrf2 and HO-1, suggesting a weakening in the protection exerted by the Nrf2/HO-1 system [31].

A goal of future research may be to spatially and temporally modulate the molecular pathways involved in the potentiation of the antioxidant responses versus oxidative stress. It should be noted that high levels of SMO can be neurotoxic in the brain, not only generating ROS (*i*.*e*., H_2_O_2_) but also producing spermidine and reactive aldehydes such as 3-AP [32]. In this respect, increased brain polyamine catabolism, with concomitant generation of toxic metabolites (*e*.*g*., 3-AP), has been observed after traumatic brain injury, silent brain infarction and stroke [33,34]. Interestingly, it has been hypothesized that agents that can chemically neutralize reactive aldehydes should demonstrate neuroprotective synergic actions with the antioxidant response [11]. To this end, hydroxylamines have been proposed as aldehyde-trapping agents both in an *in vitro* model of neurodegeneration induced by the reactive aldehyde 3-AP and in an *in vivo* rat model of hippocampal neurodegeneration [11]. However, in murine keratinocyte cells, spermidine and spermine were able to trigger an antioxidant response, increasing the expression of phase 2 genes through the activation of the Nrf2-ARE pathway by acrolein, a 3-AP-derived aldehyde [35].

Thus, a treatment strategy may be on the one hand to act at the NMDAR level, by using specific inhibitors, and/or at the SMO level (either upstream using CHL or downstream by inhibiting 3-AP and/or ROS), and on the other hand to potentiate the antioxidant responses using Nrf2-activating compounds. Currently, the Nrf2-ARE pathway is a high-value therapeutic target for several neurodegenerative diseases (e.g., Alzheimer’s disease, Parkinson’s disease, amyotrophic lateral sclerosis, Huntington’s disease, and multiple sclerosis), and numerous cell-based and in silico high-throughput screens have identified novel Nrf2-activating compounds [36]. In this respect, dimethyl fumarate (DMF) has been found recently to attenuate neurotoxicity in SH-SY5Y cells and in an animal model of Parkinson’s disease by enhancing Nrf2 activity [37]. Furthermore, DMF has been proposed to be beneficial for the treatment of neurodegenerative diseases, such as HANDs [7,38]. In particular, monomethyl fumarate (MMF), an active DMF metabolite *in vivo*, blocked ARV-induced ROS generation and neuronal damage/death, enhancing the endogenous antioxidant responses *in vitro* [7]. As recently suggested, an interesting new twist to Nrf2-dependent therapeutic approaches is that not only the pharmacological target but also the cell type targeted may be relevant [36]. Finally, with respect to Nrf2 overexpression, neurological disorders appear as promising targets for gene therapy [39].

## Conclusions

Nuclear Nrf2 accumulation and activation of ARE-driven gene expression were induced by Tat exposure in human neuroblastoma cells and seem to be regulated at multiple levels by a coordinated process involving NMDAR, SMO activation, and ROS production. Despite the need for more studies, especially in normal neuronal cells, these findings provide evidence of a primary molecular antioxidant response and, as such, may help further our understanding of the mechanism by which Nrf2 is an important target for protection against HANDs and other neurodegenerative diseases associated with HIV infection.

## Abbreviations

3-AP: aldehyde 3-aminopropanal
ARE: antioxidant-response element
ARV: antiretroviral
CAT: catalase
CHL: Chlorhexidine digluconate
DMF: dimethyl fumarate
EpRE: electrophilic response element
GAPDH: glyceraldehyde 3-phosphate dehydrogenase
H_2_O_2_: hydrogen peroxide
HAND: HIV-associated neurocognitive disorders
HIV: human immunodeficiency virus
NQO1: NAD(P)H:quinone oxidoreductase type 1
HO: heme-oxygenase
MMF: monomethyl fumarate
NAC: N-acetylcysteine
NMDA: N-methyl-D-aspartate
NMDAR: NMDA receptor
Nrf2: nuclear factor erythroid 2-related factor 2
ROS: reactive oxygen species
SMO: spermine oxidase
SOD: superoxide dismutase
Spm: spermine
